# Deep Brain Stimulation Salvages a Flourishing Dental Practice: A Dentist with Essential Tremor Recounts his Experience

**DOI:** 10.7759/cureus.840

**Published:** 2016-10-22

**Authors:** Guy Giacopuzzi, Melanie Lising, Casey H Halpern

**Affiliations:** 1 Lake Arrowhead Medical Center, Lake Arrowhead, CA, USA; 2 Department of Neurology, Stanford University School of Medicine; 3 Department of Neurosurgery, Stanford University School of Medicine

**Keywords:** bilateral, deep brain stimulation, essential tremor, frameless stereotaxy

## Abstract

In recounting his experience with deep brain stimulation (DBS), a practicing dentist challenged with long-standing bilateral essential tremor of the hands shares insights into his diagnosis, treatments, and ultimately successful DBS surgery at Stanford University Medical Center, CA, USA. Now nearly one year after his surgery, his practice continues to flourish and he encourages others in his profession to consider the possibility of DBS as a definitive treatment for tremors of the hand, which may salvage their practice.

## Editorial

It started over 20 years ago when attending a staff lunch. An internist colleague noticed my left hand tremor as I ate. He held my hand, let it go and asked if my father had a tremor. I replied in the affirmative. He quickly made the diagnosis of essential tremor (ET), and I began an odyssey that almost led to the demise of my profession as a dentist.

At my first neurological exam, I learned that ET occurs in nearly 30% of the population, is often benign, and can affect the limbs, head, neck, and voice [1]. Tremor severity can be mild, where a person learns to live with it, and many are never diagnosed. My father and grandfather were in this category. My tremor, however, involved both of my hands and was progressing. I became concerned about the future of my dental practice and sought a second opinion from a movement disorder specialist.

For the next several years I used a “four-drug cocktail” (propranolol, nadolol, primidone, and gabapentin). Maintaining my ET with these four drugs was not without side effects, most noticeably balance impairment and the feeling that “I’m on something.” These side effects were tolerable until 2014, when I developed Raynaud’s phenomenon and tinnitus, which, together with having ET, caused me to contemplate the end of my career.

As a general dentist in private practice for 35 years, I focus on implants and endodontics. I love my work and giving up my practice at the age of 61 was not in my plan. Around that time, a ‘newsflash’ on my car radio mentioned an experimental treatment for ET called Magnetic Resonance-guided Focused Ultrasound. Stanford University was a site in the multicenter randomized controlled clinical trial for ET and the closest in proximity to me [2]. Unfortunately, after a few surveys and phone calls, I soon realized this ablation treatment was only offered unilaterally. As my tremor was bilateral, significantly impairing my ability to perform procedures, I wanted a treatment that could improve both hands. I was then directed by the Stanford University research coordinator to consider deep brain stimulation or DBS. I had known about it for years but had expected to retire before needing it. She suggested that I watch a video and contact her if I was interested in a consultation.

**Video 1:** 1:04:04 / 1:13:30 Deep Brain Stimulation for Essential Tremor and Vocal Tremor Dr. Casey H. Halpern describes deep brain stimulation surgery, its indications, well-described results, and future promise for essential tremor.

The video immediately struck a chord with me. It described a surgery using a familiar modality—“image-guided” navigation. I was introduced to the concept 15 years earlier at a dental implant convention, where I had seen a surgical navigation system for image-guided implantology. It involved taking a cone-beam computerized tomography scan of a patient’s facial structures using radiographic markers or “fiducials” to provide the computer with a reference point. The surgical planning of the implant is then performed on the computer. The handpiece also has a set of fiducials; the computer, via an intraoperative sensor now “knows” where the patient’s anatomy is, where the drill is, and aligns them. The surgeon then positions the drill on the computer screen for the osteotomy—all while assessing the screen for proper alignment and depth. In the video, Dr. Casey Halpern, a DBS neurosurgeon at Stanford, explained DBS using the same navigation technology known as frameless stereotaxy [3].

Intrigued, I scheduled a consultation with Dr. Halpern. Many patients’ quality of life may be robustly improved with unilateral treatment [4]. However, performing dental implants requires the precision of both hands to optimize patient safety and outcomes. After this consultation, I made up my mind. I wanted this. I was tired of the drugs, the side effects, and I wanted my “normal life” back, or at least a life where I could practice dentistry with manageable tremors while maintaining excellent outcomes. I underwent a workup and testing and repeated the diagnostic evaluation. The neurologist I met with at Stanford, Dr. Melanie Lising, told me not every patient can discontinue medications after DBS due to residual tremor. Dr. Halpern also discussed the surgical risks, which were not minimal, but also not significant enough to deter me.

If you are an ET patient *and* dentist, preparing for surgery can be problematic. Because the tremor needs to be identifiable, the procedure is ideally performed on awake, unmedicated patients. When the electrodes are in place, intraoperative test stimulation optimizes placement. This means that patients must be weaned off medications as early as one month before surgery, and I could not work while off the medications. Thus, I scheduled the surgery for just after the winter holiday, and I took a few more days off just prior to surgery. This gave me three weeks off all the medications.

I was comfortable during the surgery. Dr. Lising assisted during the microelectrode testing and a patient advocate, who was also a massage therapist, was present to ensure that I was pain-free. The frameless stereotaxy approach allowed my head to be free and mobile for the surgery, unlike traditional stereotactic frames that are bolted to the operating room table [3].

The postsurgical time was somewhat painful but well addressed with analgesics. After one night in the hospital, I was discharged about noon the following day. The pain intensity diminished steadily, and a few days later I was using the narcotics only at night. I did notice significant fatigue. One week after surgery I returned to work, fully capable, but expectedly tired by 6 pm. During this time, the generator was off, and I was back on drugs. There was a “honeymoon” effect postoperatively where my tremor was undetectable for about one week.

My initial programming appointment finally arrived, and I again discontinued all of my medications. I didn’t realize this would entail a full day of electrode testing (eight total, four on each side), balance testing, programming, and taking the DBS “out for a spin.” The most efficacious electrodes were chosen for initial use and parameters were set using a device that sits on your shoulder and communicates with the generator transcutaneously. I received a remote control device able to turn the generator on and off, thus allowing me to vary the voltage. The implantable pulse generator, including the battery pack, lasts about three to four years [5]. Replacing it is an outpatient, local anesthesia procedure. Turning it off at night and when it’s not needed extends the battery life [5]. After this long appointment, I returned home with an optimized system and the following day I went to work.

There were a few side effects. I had difficulty saying certain words, but I’m the only one who appeared to notice. Otherwise, it worked very well. Off medications entirely, I performed three implant surgeries the first week after I was “turned on.” By the third day, I actually* forgot I had a tremor. *As my left hand was the worst, the improvement there was the most pronounced. I found myself doing things with my left hand I hadn’t done in 10 years, and I was able to do them faster than 20 years previously. Off the medications, I felt generally better, and I certainly had lost touch with what felt “normal,” having been on medications for a long time. The associated fatigue and occasional dizziness disappeared and the Raynaud’s phenomenon greatly improved. I still have tinnitus.

I returned for further programming once and Dr. Lising was able to “dial out” my speech problem. She also added two programs and changed electrode configurations on each side, leaving me in “bipolar mode.” I never realized how flexible and titratable the DBS system would be—now that it’s implanted, it’s more like medication than a surgery to recover from.

The decision to have a DBS system installed is not insignificant. Most dentists with ET eventually quit their practice, with or without medication. Many choose academic dentistry when ET progresses. But even after 36 years, I am not tired of dentistry. I actually feel ‘called’ to do what I do. Dentistry is not the only reason I wanted DBS. Having steady hands is nice for restoring old radios, working on my antique car, and tying flies for fly-fishing. 

Since implantation, a few unexpected things have happened. My patients have been overwhelmingly positive to my surgery and very thankful that I am “going to continue to be their dentist.” Only a few were even aware of my tremors. Several dentist colleagues have also “confessed” to me that they have tremors. Virtually all are undiagnosed, and I always encourage them to connect with a movement disorder neurologist or DBS neurosurgeon to consider every available option [6]. At the moment, no practitioner-based support groups exist. Though there are support groups for ET, “being a dentist” would be difficult to discuss. I’m now considering beginning an online forum for this purpose. 

Although Dr. Halpern informed me that he had seen positive effects of DBS in ET patients with professions demanding fine motor control, he had never performed DBS on a practicing dentist. That surprised me. I believe there are others who don’t want to quit clinical practice. Are they just too afraid of brain surgery? I don’t know. But I have my life back, and I’m incredibly grateful for the relief this treatment has provided me, my patients, and my practice.

## References

[1] Ho AL, Erickson-Direnzo E, Pendharkar AV, Sung CK, Halpern CH (2015). Deep brain stimulation for vocal tremor: a comprehensive, multidisciplinary methodology. Neurosurg Focus.

[2] (2016). ExAblate transcranial MR guided focused ultrasound for the treatment of essential tremors. http://clinicaltrials.gov/ct2/show/NCT01827904.

[3] Holloway KL, Gaede SE, Starr PA, Rosenow JM, Ramakrishnan V, Henderson JM (2005). Frameless stereotaxy using bone fiducial markers for deep brain stimulation. J Neurosurg.

[4] Huss DS, Dallapiazza RF, Shah BB, Harrison MB, Diamond J, Elias WJ (2015). Functional assessment and quality of life in essential tremor with bilateral or unilateral DBS and focused ultrasound thalamotomy. Mov Disord.

[5] Halpern CH, McGill KR, Baltuch GH, Jaggi JL (2011). Longevity analysis of currently available deep brain stimulation devices. Stereotact Funct Neurosurg.

[6] Zesiewicz TA, Elble RJ, Louis ED, Gronseth GS, Ondo WG, Dewey RB Jr, Okun MS, Sullivan KL, Weiner WJ (2011). Evidence-based guideline update: treatment of essential tremor. Report of the quality standards subcommittee of the American Academy of Neurology. Neurology.

